# Targeted Cancer Therapy: Vital Oncogenes and a New Molecular Genetic Paradigm for Cancer Initiation Progression and Treatment

**DOI:** 10.3390/ijms17091552

**Published:** 2016-09-14

**Authors:** Rudolph E. Willis

**Affiliations:** OncoStem Biotherapeutics LLC, 423 W 127th St., New York, NY 10027, USA; rwillis@oncostembio.com; Tel.: +1-484-273-8367; Fax: +1-267-886-8339

**Keywords:** oncogenes, oncogene addiction, carcinogenesis, transcription factor, cancer genome, gene fusion, cancer genetics, cancer stem cell, targeted cancer therapy, personalized medicine

## Abstract

It has been declared repeatedly that cancer is a result of molecular genetic abnormalities. However, there has been no working model describing the specific functional consequences of the deranged genomic processes that result in the initiation and propagation of the cancer process during carcinogenesis. We no longer need to question whether or not cancer arises as a result of a molecular genetic defect within the cancer cell. The legitimate questions are: how and why? This article reviews the preeminent data on cancer molecular genetics and subsequently proposes that the sentinel event in cancer initiation is the aberrant production of fused transcription activators with new molecular properties within normal tissue stem cells. This results in the production of vital oncogenes with dysfunctional gene activation transcription properties, which leads to dysfunctional gene regulation, the aberrant activation of transduction pathways, chromosomal breakage, activation of driver oncogenes, reactivation of stem cell transduction pathways and the activation of genes that result in the hallmarks of cancer. Furthermore, a novel holistic molecular genetic model of cancer initiation and progression is presented along with a new paradigm for the approach to personalized targeted cancer therapy, clinical monitoring and cancer diagnosis.

## 1. Introduction

Cancer is a molecular genetic disease [1,2]. More specifically, it is a manifestation of the dysfunctional regulation of the normal genomic processes responsible for cell differentiation, growth, replication and cell death. More than one hundred years ago, Boveri theorized that the etiology of the cancer process lies buried within the confines of our chromosomes [3]. We now know that the problem lies specifically within our genes. Older vague paradigms of the cancer process need to be replaced with new evidenced-based models to explain the initiation and progression of cancer. Such models will not only reveal deep insights into the normal mechanisms of human gene regulation, but will enhance our capacity to identify the true initiators and drivers of the oncogenic process [4]. Such models will shed light on the vital oncogene targets that are the Achilles heel of the cancer cell. This will lay the foundation for truly personalized cancer therapy.

A suitable molecular genetic model for cancer must not only provide a reasonable explanation and description of the genomic events manifested in the cancer cell, but also should provide the etiological basis for genetic intratumor heterogeneity and the hallmarks of cancer [5,6]. Paramount to this discussion is the crucial relevance of the seemingly inherent genetic instability of cancer and its relationship to cancer initiation and progression [7]. Such genetic instability provides a reasonable explanation for a cancer cell’s ability to accumulate the innumerable genetic alterations typically seen in virtually every cancer. However, in addition, it provides the explanation for the sustained increase in mutation rate as the cancer cell proceeds from its initiation event through its progression. There are a myriad of genetic abnormalities within the cancer cell. Therefore, it is tremendously important that there is a clear understanding of the nature of these molecular genetic changes and their relevance to the design of curative targeted cancer therapy. Which molecular genetic alterations are collateral bystander phenomena without significant therapeutic relevance? Which of these alterations play a crucial role in cancer progression? Additionally, which of these alterations represents the vital oncogenes that not only initiate the cancer process, but are mandatory for the cancer cell’s survival? To establish the validity of the answers to such questions, we will need to review some pertinent information concerning carcinogenesis, certain aspects of the cancer genome, cancer cell biology and the nature of the mutations commonly found within the cancer cell. This will lay the groundwork for the construction of a holistic molecular genetic model for cancer initiation and progression.

## 2. Carcinogenesis

### 2.1. Facts and Theories

Traditionally, we have invoked the potential contribution of abnormal physiological processes in our attempt to explain the etiology of the neoplastic process. A typical example is the role attributed to excessive inflammation [8]. Only recently have we begun to understand the molecular genetic foundation for such beliefs. Data readily demonstrate that some driver oncogenes play a symbiotic role in inflammation and tumor progression [9]. However, it is proposed here that the molecular events in the inflammatory process that appear to cause cancer development are in reality a component of the progressive portions of the neoplastic process perpetrated by oncogene drivers and are not a part of the foundational sentinel events that initiate carcinogenesis.

If we wish to understand the true nature of the role of molecular genetics in carcinogenesis, we must have knowledge of some relevant cellular and genomic physiological processes. Cancer develops by a multi-step process [10]. Traditionally, we have divided the cellular pathophysiological process into categories [11,12]. The initiating event is irreversible. It is assumed that this is the result of a stable cellular change that is the result of the initial carcinogenic event. This is the first step in carcinogenesis. Critical gene mutation events occur, which predispose the affected cell and its progeny to subsequent neoplastic transformation. The second broad category is best described as a progression of the neoplastic process [13]. During progression, the process becomes increasingly irreversible with the onset of genetic instability, a higher growth rate, changes in biochemical and metabolic processes and morphological changes. Additional mutations result in the rise of increasingly heterogenetic malignant sub-populations with increasing survival capacity. A host of varied mutation types can be found within the cancer cell. These may consist of point mutations, insertions, deletions, inversions, amplifications and gene translocations. However, it is proposed here that the significance of a given type of mutation lies within a hierarchy. Furthermore, during this process, chromosomal abnormalities may appear. Here, we are specifically concerned with the mutation events that occur within somatic cells. The gene mutation theory of cancer maintains that it is the somatic gene mutations that form the basis of neoplastic transformation [14]. In essence, this theory states that cancer results from a single somatic cell that has accumulated multiple DNA mutations and that cancer is a disease of cell proliferation caused by mutations in genes that control proliferation and the cell cycle.

The efficient ability of the cell to recover from damaging gene mutations inevitably determines the cell’s fate. DNA damage may result from intrinsic insults, such as base pair mismatching during DNA replication, collapse of replication forks or even as a result of reactive oxygen species produced during abnormal cellular metabolism. However, the greatest DNA damage often occurs as a result of extrinsic insults, such as exposure to ultraviolet light, ionizing radiation or environmental mutagens. The most harmful form of DNA damage results in double-strand breaks [15,16]. Unrepaired double-strand DNA breaks result in severe consequences, including genomic instability and cell death. Mammalian cells are equipped with two methods to achieve the repair of double-strand DNA breaks. Homologous recombination achieves this by copying intact information from an undamaged homologous DNA template. Non-homologous end joining rejoins broken ends regardless of sequence. If there is a failure of this process, significant genomic instability and a predisposition to further DNA damage results. It is proposed here that poorly-corrected double-strand DNA breaks are mandatory for the initiation of the abnormal genomic process that results in the production of vital oncogenes, which then orchestrate neoplastic transformation [16].

### 2.2. Cancer Stem Cells

The human body is highly compartmentalized consisting of different organs and tissues. Normal tissues require structure. They require an integument composed of tissue cells characteristic of a particular organ. There is a common cellular mechanism that maintains the constant population of cells within any given tissue. This process is highly conserved. It is a process that adheres to closely-regulated steps [17]. It is the organ’s stem cells that serve as the seed that leads to organogenesis. This is true of every organ in the human body. It has been suggested that the cancer process is a form of dysfunctional organogenesis resulting from the loss of gene regulatory control [18].

The work by Scott was one of the earliest studies demonstrating the possibility that the initiation of carcinogenesis could arise in stem cells as a result of chemical or physical carcinogens or certain viruses [19]. In addition, these same initiated stem cells may undergo a promotion process resulting in complete neoplastic transformation. This concept coincides at the cellular level with the classical two-stage evolution of neoplastic transformation. If we further extend this concept at a molecular genetic level, it follows that the initiation event within these stem cells is equivalent to the sentinel mutation of vital oncogenes, and the promotion process equates to the progression of the neoplastic process as a result of the increased mutational state resulting from the dysfunctional genomic effects of such vital oncogenes.

A tremendous amount of data is now evident indicating that cancer consists of a hierarchy of functional tumor cells supported by the presence of treatment-resistant cancer stem cells. This concept is known as the cancer stem cell theory [20,21,22,23,24,25,26]. It is believed that this subpopulation of cells within a variety of cancer types consists of self-renewing cells with tumorigenic potential lacking in the remaining cells of the tumor. Furthermore, studies suggest that the regulation of the pathways responsible for stem cell renewal has been violated within this subpopulation of cells. Riggi has demonstrated that the fused transcription activator *EWS-FLI1* vital oncogene is capable of transforming primary bone marrow-derived mesenchymal progenitor cells, leading to the formation of tumors that display all of the hallmarks of Ewing’s sarcoma [27]. Our current therapies, which are designed to eradicate the proliferating cells within a cancer, often fail. Perhaps this is a consequence of the presence of the infrequently-replicating cancer stem cells that drive the tumorigenic process. It has been suggested that the only effective and curative way to approach cancer therapy is to direct targeted therapy against this subpopulation of cells [28,29,30,31,32].

## 3. The Cancer Genome

### 3.1. Transcription Activators

The process that controls the normal function of the genome within the cancer cell is drastically altered. It is more than evident that transcription factors play a crucial role in normal gene regulation [33]. Since transcription factors regulate virtually every fundamental developmental and homeostatic cellular process, it is expected that acquired structural defects within this subset of cellular genes play a crucial role in carcinogenesis. Transcription factors are composed of a group of gene regulatory proteins with a variety of physiological functions that are functionally connected to signal transduction pathways [34]. For example, acetyltransferases and methyltransferases act on specific targets that facilitate or hinder gene activity. Other transcription factor proteins function during the chromosomal modification needed to allow gene transcription. However, gene expression would not occur without normally functioning transcription activators. Transcription activators are a unique subset of the transcription factor proteins that recognize and bind to sequence-specific regulatory enhancer or suppressor sequences in DNA and subsequently recruit the components of the DNA transcription apparatus mandatory for the initiation of DNA transcription [35,36].

Some transcription activators are essential for cellular proliferation [37]. *E2F1-3* transcription activators regulate a cell’s normal progression through the G1/S transition during the cell cycle. This is the proto-oncogenic equivalence of the expected activity demonstrated by a vital oncogene [4]. The role of transcription activators in neoplasia has been evident for some time [38]. *SOX9* is upregulated commonly in colorectal cancer. Furthermore, strong *SOX9* expression is an independent indicator for an adverse prognosis in colorectal cancer [39]. Jiang demonstrated the upregulation of *SOX9* expression in lung adenocarcinoma and its direct effect on cell growth through its effect on the expression of cell cycle regulators [40]. Likewise, Huang demonstrated the role of *SOX9* in the initiation of prostate cancer [41]. Furthermore, Chen’s in vitro study reveals direct evidence that *SOX2* regulates a transcriptional network of oncogenes and affects tumorigenesis in lung cancer stem cells [42]. In addition, it appears that transcription activators may regulate the gene expression of the recombination activating gene 1 (*RAG1*) in cancer cells [43]. This is a crucial concept that plays a role in the molecular genetic carcinogenesis model developed here.

### 3.2. Gene Recombination

*RAG1* and *RAG2* normally function in the process that leads to functional immunoglobulin and T cell receptor gene assemblage from their respective multiple gene coding segments. Evidence of the direct involvement of the *RAG* gene in gene translocations resulting in gene fusions comes from studies in mice [44,45,46,47,48]. Normal gene recombination is directed by specific recombination signal sequences adjacent to each coding segment [49]. V(D)J recombination is initiated by the introduction of a double-strand break between the recombination signal sequence and the neighboring coding DNA. Hiom has shown that this process can be defective in certain environments, resulting in the inappropriate diversion of V(D)J rearrangement to a transpositional pathway that leads to unwanted and defective gene translocations [50]. If such intrinsic injurious events occur, this provides the opportunity for illegitimate nonhomologous end-joining at the sites of double-strand breaks mistakenly produced by aberrantly-functioning recombination activating genes. Such a phenomenon would be analogous to extrinsic DNA insults from radiation resulting in double-strand DNA breaks that lead to gene rearrangements as a result of attempted repair by nonhomologous end-joining [51]. Illegitimate recombination mechanisms have been identified in solid tumors [52,53]. We can envision the rampant effects of uncontrolled recombination activating genes in the cancer cell. Such an event would explain the previously poorly-understood cancer phenomenon, chromothripsis, that results in massive focal gene rearrangements in some cancer cells [54,55,56,57,58].

### 3.3. Transposons

The other intrinsic event that involves double-strand DNA breaks is a result of transposable DNA sequences, called transposons [59]. The defining property of this genetic element is its ability to move from one position to another in the genome. All forms of this entity achieve this by introducing staggered breaks in DNA by an associated enzyme, transposase. Some replicate, and some do not, during this process. Transposable elements probably play a major role in genomic evolution and the rearrangement of genomes [60]. Genome sequencing reveals that these entities constitute a large fraction of eukaryotic genomes. They may occupy nearly 50% of the human genome [61,62]. Active transposable elements are extremely mutagenic. Their effect on the protein-coding genes that they often target can cause insertions, chromosomal breakage, illegitimate recombination and genome rearrangements. The *APC* gene is a tumor suppressor gene associated with the development of sporadic colon cancer [63]. Miki demonstrated the disruption of the *APC* gene caused by somatic insertion of a transposon in a colon cancer [64]. Work by Iskow also revealed novel transposon insertions at high frequency in human lung cancer genomes [65].The implication is that disruption of the normal cellular mechanisms that suppress transposon activity may facilitated the mutation process that drives the progression of the cancer [66].

## 4. Mutations in Cancer

### 4.1. The Genomic Landscape

The one prevailing fact about cancer is that mutations are an integral part of malignancy. Loeb declared that cancers actually exhibit a mutational phenotype [67,68]. This concept has implications for the validity of a holistic molecular genetic model of carcinogenesis. It follows that malignancies during cancer progression should be capable of a greater mutation rate compared to normal tissues. Mutations that subsequently directly impact on genetic stability are responsible for the mutator phenotype. The resulting hyper-mutation state should increase the efficiency of additional mutations beneficial to cancer cell survival. Finally, both clonal and random mutations should be produced as the cancer progresses, resulting in the appearance of driver mutations and insignificant passenger mutations.

Somatic mutations in the cancer cell encompass several types of DNA sequence changes [69]. These include base substitutions; insertions and deletions of DNA segments; copy number changes; and gene rearrangements resulting in inversions or translocations. Despite the mutation heterogeneity seen across tumor types, within a particular tumor and in a single individual there are emerging patterns to these somatic mutations [70]. Improved gene sequencing techniques are allowing us to visualize the genomic landscapes of cancers [71,72]. Kandoth presented and analyzed the recent data generated by the Cancer Genome Atlas project [73]. The sequencing and analysis only focused on point mutations and small insertions or deletions in 3281 tumors across 12 tumor types. The number of so-called driver mutations required during oncogenesis was relatively small. More significantly, mutations in transcription factors showed tissue specificity. Other than this fact, the data essentially said nothing about the potential mechanisms involved in the initiation and progression of these cancers. Futreal conducted a census from the literature of genes that are mutated and causally implicated in cancer development. The most common mutation class among the known cancer genes was a chromosomal translocation that created a chimeric gene or apposed a gene to the regulatory element of another gene. Many of the newly-identified cancer genes were found in leukemias, lymphomas and sarcomas. These genes were usually altered by gene translocation [74].

### 4.2. Gene Fusions

One of the first gene fusions identified results from a translocation [75]. The Philadelphia chromosome commonly present in chronic myelogenous leukemia results from the interchange between the end of the long arm of chromosome 9 and the long arm of chromosome 22 [76]. It is now clear that gene fusions play an important role in the initial steps of carcinogenesis [77]. More than 300 gene fusions involving more than 300 different genes have been identified [78]. The available data show that gene fusions occur in all malignancies. Furthermore, gene fusions have been shown to be present in the stem cell compartment of early progenitors in acute leukemias, as well as in progenitor cells that give rise to liposarcoma [79,80].

The greatest challenge faced in the effort to eradicate cancer lies in a misunderstanding of the nature of the true oncogenic initiators of carcinogenesis and the sustainers of subsequent neoplastic progression. The past and current research and therapeutic focus have been placed on pursuing the so-called ‘drivers’ of the cancer process [81]. As a result, current research is bogged down in a tug-of-war between the identification of causative genetic changes in the cancer cell, as opposed to those that are simply peripheral passenger genetic changes that subsequently participate in the cancer process, but in reality, are merely a consequence of the aftermath of the initial few sentinel events [82,83]. Driver mutations participate in the consequences of the cancer process. They are not initiators of the sentinel events that contribute to the irreversibility of cancer progression. This explains the inevitable development of resistance to therapies targeting just these entities [84]. At an even lower level of significance, passenger mutations make their appearance. This is also a byproduct of the cancer process. They may be deleterious, but do not drive the cancer process either [83]. Current research has attempted to circumvent our confusion by purely analytical and computational methods [85,86,87,88,89]. Furthermore, it is clearly evident that the continued attempt to simply tabulate mutational changes in cancer through genomic methods cannot provide the right answers to the kind of questions we need to ask to identify the vital oncogenic targets for therapies that will finally prove to be definitive and utterly effective [90]. We need a model.

## 5. Leukemia: A Model for Carcinogenesis

Does the nature of leukemia open the door to an understanding of the cancer process? Does the investigation of the cell biology and the molecular genetics of this disease tell us something important about carcinogenesis? Unlike solid tumors, it has fluidity. Physically, it is more malleable and more accessible for intense scrutiny [91,92]. The work by Bonnet and Dick first demonstrated the potential origination of the leukemic cancer cell from a normal leukemic stem cell or a progenitor cell [93]. Acute leukemias are clonal neoplasms that arise from the development of genetic alterations. This results in the arrest of the normal differentiation process initiated by stem cells and the production of immature cancer cells in the blood and bone marrow. In acute myeloid leukemia, more than 50% of adult patients have cancer cells that contain non-random chromosomal abnormalities, including most significantly gene translocations [94]. It is clear that the molecular genetic events involved in leukemic pathogenesis are complex [95]. However, transcription activator fusions were the first recognized somatic mutations in this disease and have been shown to initiate this disease in mice [96,97]. Furthermore, there are prognostic implications of many of the genetic changes found in acute leukemia [98,99]. A striking example is the impact of mutations involving the runt-related transcription activator gene *AML1* (also known as *RUNX1* and *CBFA2*). This gene is located on chromosome 21 and is frequently translocated with the *ETO* gene located on chromosome 8q22, resulting in the *AML-ETO* fusion protein. *AML1* mutations are associated with resistance to standard induction therapy with inferior survival for younger and older patients [100,101]. Mendler also found that the gene expression profile associated with such *AML1* mutations is very similar to that present in normal hematopoietic stem cells and progenitors [100]. Other similar types of translocations involving the *MLL* transcription activator also have a characteristic distinct gene expression profile consistent with that seen in a hematopoietic progenitor or stem cell [102,103].

Chromosomal translocations in acute leukemia often rearrange the regulatory and coding regions of a variety of transcription factor genes [104]. As a matter of fact, the most frequent targets of gene translocations in this disease are the genes that are responsible for transcription activation and its associated processes. The oncoproteins produced by this process may interfere with the normal transcriptional networks that function in concert with growth factors and their receptors and the normal transduction pathways that regulate hematopoiesis. These leukemia-associated fusion proteins have common structural and functional characteristics indicative of their ability to impart leukemic phenotypes through common modes of transcriptional dysregulation [105]. It appears that such vital oncogenes demonstrate ‘gain-of-function’ activities not shared by the constituent proteins. For example, the *AML1-ETO* fusion protein upregulates *AP-1* activity. Furthermore, the expression of *AML1-ETO* leads to increased amounts of the phosphorylated *JUN* and *ATZ* genes, suggesting an increased activity of the *MAPK* pathway, which is crucial to the cancer process in many other cancer types [105]. This vital oncogene’s capacity to orchestrate such an event may be related to its loss of the nuclear matrix-targeting signal that directs the normal *AML1* protein to the appropriate gene regulatory sites within the nucleus [106,107]. Most significantly, the fused transcription activator proteins found in leukemia are capable of inducing leukemia in mice models and in NIH3T3 cells [96,97,108]. Furthermore, Frank found that the *AML1-ETO* fusion protein not only promotes the phosphorylation of *JUN* and transcriptionally activates *AP-1* responsive promotors, but also promotes cellular transformation. This is reflective of the gain-of-function properties associated with this oncogene [108]. In summary, it appears that the fused protein products of transcription activators have unique qualities that are important in the cancer process.

## 6. Carcinogenesis in Solid Cancers

### 6.1. Cancer Stem Cells in Solid Tumors

The traditional concept that neoplasms result from an exogenous or endogenous event that induces critical mutations within a normal cell is well accepted. The assumption is that these mutations lead to a transformation of that normal cell to one that is more primitive with a new proliferative capacity and is capable of expanding clonally while inherently self-sustaining. The only ‘normal’ cell that fulfills such criteria must have the innate characteristics of a stem or progenitor cell. Adult stem cells normally occupy discrete niches within every organ. In every tissue, they provide the normal internal repair and replenishing mechanism needed for organ function. These cells have a long life span and produce progeny that are multipotent with the capacity to recapitulate the whole range of cell types normally found within a specific organ. The tissue stem cell’s very longevity and self-renewal ability places it at risk for exposure to the initial crucial genetic event that starts neoplastic transformation. These concepts explain why a malignant tumor may resemble a new organ composed of abnormally-differentiated cells with both genotypic and phenotypic diversity. The doubts regarding the existence of cancer-initiating cells in solid tumors are considerably less as a result of accumulating data that have shed increasing light on the presence of a cancer stem cell subpopulation that probably participates in an important aspect of the cancer process in all solid tumors [109,110]. The only remaining controversy is whether or not the cancer initiation cell arises from crucial mutations in a tissue stem cell, its progenitor cell or both.

Investigations of sarcomas have provided the foremost scientific data on cancer stem cells in solid tumors. Suva isolated a subpopulation of CD133+ tumor cells that displayed the capacity to initiate and sustain tumor growth through serial transplantation in non-obese diabetic/severe combined immunodeficiency mice, re-establishing at each in vivo passage the parental tumor phenotype and hierarchical cell organization [111]. The synovial sarcoma cell lines established by Naka expressed the stem cell marker genes *OCT3/4*, *NANOG* and *SOX2*. Furthermore, most significantly upon silencing the *SS18-SSX* fused transcription activator gene with sequence-specific siRNAs, these cells exhibited morphological transition from spherical growth in suspension to adherent growth in a monolayer, additional expression of later mesenchymal lineage genes and broader differentiation potentials into osteocytes, chondrocytes and adipocytes [112]. Stratford isolated a subpopulation of liposarcoma cells that expressed both aldehyde dehydrogenase and CD133 capable of self-renewal and the capacity to generate tumors in vivo from as few as 100 injected cells [113].

Similarly, a host of other solid tumors have been investigated in an attempt to identify that subpopulation of cancer cells potentially responsible for cancer initiation. Al-Hajj grew human breast cancer cells in immunocompromised mice and was able to distinguish tumorigenic from non-tumorigenic cancer cells based on cell surface marker expression. Tumorigenic cells were CD44 positive and CD24 low or negative. As few as 100 cells with that phenotype were able to form tumors in mice; on the other hand, it required tens of thousands of cells with alternative phenotypes to do so [114]. The tumorigenic subpopulation could be serially passaged. Each time, cells within this subpopulation generated new tumors containing additional CD44-positive and CD24 low or negative lineage tumorigenic cells, as well as the phenotypically-diverse mixed populations of non-tumorigenic cells present in the initial tumor. Jauffret identified breast cancer stem cells with metastatic capacity and a distinct molecular signature [115]. Nearly 70% of the established cell lines contained an aldehyde dehydrogenase-positive population that displayed stem cell properties in vitro and in xenografts. Gene expression profiling identified genes known to play a role in stem cell function.

Li chose to evaluate pancreatic cancer. Like others, he utilized a xenograft model in which primary human pancreatic adenocarcinomas were grown in immunocompromised mice allowing the identification of a highly tumorigenic subpopulation of pancreatic cancer cells that expressed the cell surface markers CD44, CD24 and epithelial-specific antigen (ESA). Pancreatic cancer cells expressing the cell surface marker CD44+ CD24+ ESA+ phenotype had a 100-fold increased tumorigenic potential compared to non-tumorigenic cancer cells. The resulting tumors were histologically indistinguishable from the human tumors from which they originated. This subpopulation of highly tumorigenic cells showed the stem cell properties of self-renewal, the ability to produce differentiated progeny and an increased expression of the developmental signaling molecule Sonic Hedgehog [116]. Multiple solid tumors have been investigated in a similar manner revealing the presence of tumorigenic cancer stem cells in prostate, lung, colon as well as head and neck cancer [117,118,119,120,121]. Finally, glioblastoma appears to have a cancer stem cell hierarchy [122,123]. A subset of the cancer stem cell population in gliomas directly affects clinical outcome [124].

### 6.2. Fused Transcription Activators in Solid Tumors

Initially, it was believed that solid tumors did not harbor significant gene fusions related to the cancer process. However, next generation gene sequencing advances and accessibility to large series of annotated clinical material has completely altered that belief. This has also clarified the nature of these fusion events [125,126,127]. For example, the *MYB* gene, a transcription activator, has been identified in gene fusion events in both breast and head and neck cancers [128].

The mere presence of such fused genes is insufficient. A causal and functional relationship to the cancer process in solid tumors needs to be evident, as well. Naka’s earlier work revealing the inhibitory effect of siRNA on *SS18-SSX* in synovial sarcoma stems cells is relevant [112]. Barr carefully investigated alveolar rhabdomyosarcoma, an aggressive pediatric soft tissue cancer with muscle differentiation [129]. Using mapping and cloning strategies, he identified that rearranged *PAX3* and *PAX7* genes, which encode members of the paired box transcription factor family, are juxtaposed to the *FKHR* gene, which is a transcription activator. These translocations result in chimeric transcripts that encode fusion proteins that contain the *PAX3* or *PAX7* DNA-binding domain and the COOH-terminal *FKHR* transcriptional activating domain. In transfection studies, the *PAX3-FKHR* fusion activates the transcription of reporter genes containing *PAX* DNA-binding sites and is 10- to 100-fold more potent as a transcriptional activator than the *PAX3* wildtype. Distinct gene expression signatures are associated with *PAX3-FKHR* or *PAX7-FKHR* gene fusions in rhabdomyosarcomas and determine the prognosis in this cancer [130]. Losada evaluated the consequences of the presence of the *FUS-CHOP* chimeric fusion protein in transgenic mice [131]. He introduced the *FUS-CHOP* fused gene into the mouse genome with subsequent production of the protein product. The overexpression of *FUS-CHOP* resulted in most of the characteristics of human liposarcomas, including the presence of lipoblasts with round nuclei, the accumulation of intracellular lipid, the induction of adipocyte-specific genes and a corresponding block in the differentiation program. Likewise, Riggi transformed primary mesenchymal progenitor cells into tumors resembling human myxoid liposarcoma by the insertion of the *FUS-CHOP* gene [80]. Transcription profile analysis of these tumors revealed induction of transcripts known to be associated with myxoid liposarcoma.

It appears that breast cancers are not exempt from gene fusions [132]. Somatic rearrangements are not uncommon in breast cancer. The use of paired-end sequencing strategies has revealed the presence of more rearrangements in this disease than previously thought [133,134]. Interesting gene fusions have also been identified in prostate cancer [135]. Recurrent fusions of the gene *TMPRSS2* and the *ETV1* transcription activator gene have been identified in prostate cancers by Tomlins [136]. As a result of improving techniques, an increasing number of gene regulatory fusion proteins will be identified in all solid tumors. These entities are the vital oncogenes we have so desperately sought after, and they will become the foundation of both a whole new diagnostic and therapeutic approach to cancer therapy.

## 7. Vital Oncogenes

### 7.1. Characteristics and Mechanisms of Action

Vital oncogenes are fused transcription activators originating in normal tissue stem or progenitor cells and have acquired new potent and dysfunctional gene regulatory abilities that result in the initiation, progression and the maintenance of the cancer process. Vital oncogenes are not just mutated driver cancer genes. They are involved in the initial step in carcinogenesis and the early part of cancer progression. It is proposed that vital oncogenes maintain the cancer state. In this scenario, driver cancer genes simply function as the work horses that continually manifest the characteristics of the cancer cell and the hallmarks of cancer. The *MYC* gene is a potent transcription activator proto-oncogene [137]. It is transformed into a vital oncogene when it is juxtaposed next to an immunoglobulin locus [138].

The *PAX3-FKHR* fusion gene results from a translocation between chromosomes 2 and 13 in rhabdomyosarcomas. The amino-terminal paired box and homeodomain DNA-binding domains of PAX3 are fused in frame to the COOH-terminal regions of the chromosome 13-derived *FKHR* gene. Fredericks demonstrated that the fused protein of this vital oncogene is highly expressed in the nucleus, displays altered binding of a reference *PAX* DNA target and can excessively activate transcription relative to the wildtype *PAX3* [139]. Bennicelli attributes such ‘gain-of-function’ directly to the new structural properties of the chimeric protein produced by *PAX3-FKHR* [140]. The *EWS-FLI1* fusion gene produced from a translocation of chromosomes 11 and 22 results in the juxtaposition of the *FLI1* transcription activator next to the EWS gene in Ewing sarcoma. This vital oncogene has gained the ability to alter chromatin by depleting nucleosomes at targeted gene expression sites. The capacity to alter its normal gene-targeted sites ultimately leads to transcriptional dysregulation [141]. Furthermore, the *EWS-FLI1* fusion gene is directly responsible for the deregulation of *GLI1*, the critical effector of Hedgehog signaling [142,143]. The Hedgehog pathway is activated in many cancers. The oncogenic potential of this pathway is mediated by increasing the activity of the *GLI* family of transcription activators. Other vital oncogenes, such as *AML1-ETO*, are capable of inducing the *WNT* signaling pathway, another important pathway activated in cancer [144]. Vital oncogenes should have some effect on the cell cycle. Although the *SSX* family of genes normally functions as suppressors, when *SSX1* is fused to *SYT1* in the *SYT1-SSX1* gene in synovial sarcoma, the fused gene is capable of increasing the expression of both cyclin A and D1. This suggests a link between this oncogene and the cell cycle machinery [145]. Willis has proposed a theoretical mechanistic model that describes another potential function of vital oncogenes in the dysregulation of the cell cycle during carcinogenesis [4]. Vital oncogenes also appear to play a role in the development of the stem-cell-like behavior of cancer cells. Alcalay expressed *AML1-ETO* inU937 hemopoietic precursor cells and measured global gene expression using oligonucleotide chips. The fusion protein of this oncogene induced genes involved in the maintenance of the stem cell phenotype and repressed DNA repair genes that function in the base excision repair pathway. Functional studies confirmed that the ectopic expression of the oncogenic *AML1-ETO* fusion protein constitutively activated pathways leading to increased stem cell renewal (Jagged1/Notch pathway) and provoked accumulation of DNA damage. The expression of *AML1-ETO* essentially resulted in the expansion the stem cell subpopulation and the induction of a mutator phenotype [146].

### 7.2. Holistic Molecular Genetic Model for Carcinogenesis

We have reviewed some of the important molecular genetic details of the cancer cell. Mutations are the underpinnings of the cancer process. However, cancer mutations are hierarchal in importance. It follows that certain mutations are more relevant than others and that the cancer cell depends on these mutated genes for its survival. Oncogene addiction is the term that Weinstein applied to this concept [147]. Vital oncogenes may be the ultimate and ideal entity to which this concept applies. 

A self-evident major premise supporting the veracity of any molecular genetic model for carcinogenesis assumes that certain mutations in cancer are vital to its initiation and early progression. In addition, such instigating mutations must characteristically have the capacity to remain in the genome of certain cell progeny, resulting in the recapitulation of the cancer initiation process and early progression within that subpopulation of new cells. Here, it is proposed that the sentinel event resulting in the initiation of the cancer process is the externally- or internally-mediated development of double-strand DNA breaks within tissue stem cells and/or progenitor cells that allow the occurrence of gene rearrangements. This leads to the formation of oncogenic fused proto-oncogene transcription activators with novel gene activating and DNA regulatory properties.

The internal and external mediators of DNA double-strand breaks have been well established. These include: the DNA damaging effects of chemicals and viruses; the consequences of illegitimate non-homologous end joining; the consequences of illegitimate homologous recombination; illegitimate class switch recombination; hereditary mutations in DNA repair genes; the presence of fragile DNA sites; and the DNA damaging effects of radiation. Ionizing radiation can directly generate leukemic-specific fusion genes, such as *AML1-ETO* [148]. The chimeric oncogenic proteins that result from these events are dysfunctional gene regulators with the ability to reset gene promotor targets, which could lead to the activation of genes controlled by other normal transcription activators and lead to the constitutive activation of genes responsible for stem cell characteristics and transduction pathways active in cancer. The ultimate result is the production of driver oncogenes that fuel the cancer process, culminating in the manifestation of the hallmarks of cancer.

**Figure 1:**
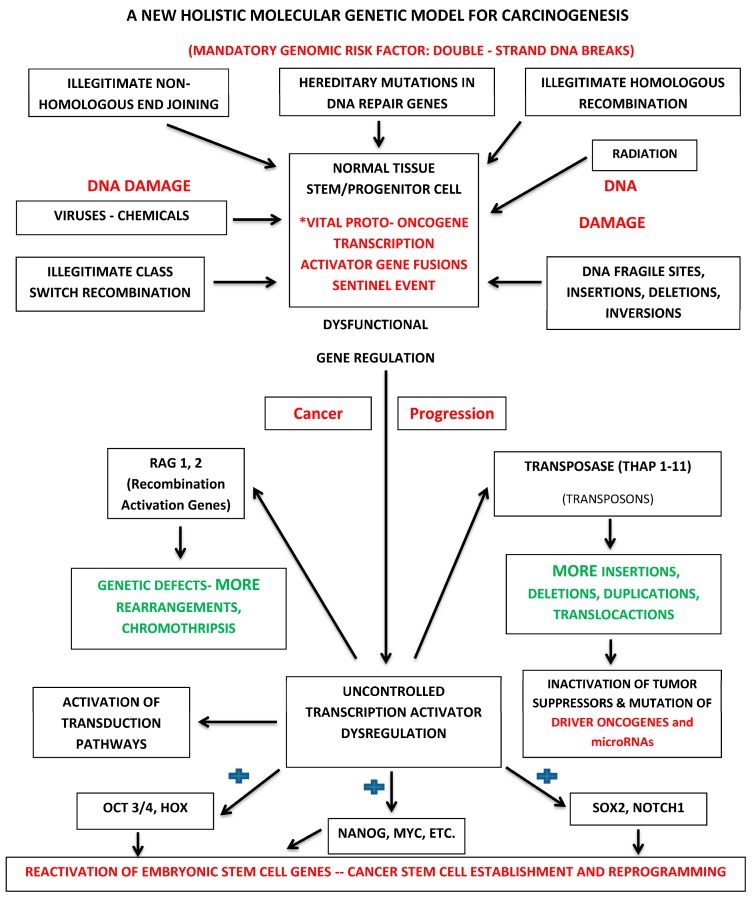
Holistic molecular genetic paradigm for cancer initiation and progression. * The sentinel event in cancer occurs when a double-strand break is introduced into DNA at the site of a proto-oncogene transcription activator within a normal tissue stem or progenitor cell resulting in the subsequent production of a chimeric oncogenic protein with novel gene regulatory properties. The blue shape (+): vital oncogenes constitutively activate stem cell maintenance genes such as *OCT3/4*, *HOX*, *NANOG*, *MYC*, *SOX2* and *NOTCH1*.

We can now construct a holistic molecular genetic model for carcinogenesis based on vital oncogenes. It is presented in Figure 1.

## 8. Implications

### 8.1. Targeted Cancer Therapy

The current approach to targeted cancer therapy focuses on so-called cancer drivers. This is equivalent to treating the symptoms, the hallmarks of the cancer process, and not the disease. Even in the presence of current targeted cancer drugs, many cancers develop resistance. Because current targets are not vital to the perpetuation of the neoplasm, a tumor will inevitably evolve feedback mechanisms that activate other pathways that will sustain it [149]. The residual cancer cell remains a cancer cell. It still harbors the vital oncogenes that probably initiate and maintain the cancer process. The other aspect of therapeutic failure involves the presence of chemotherapy resistance. It is becoming increasingly clear that the preponderance of chemotherapy resistance seen in cancer may well be the result of the resilience of the cancer stem cell [150]. Weinstein has asked how do we identify the Achilles’ heel in specific cancers, so that each patient can be treated with the appropriate molecular targeted agent [151]? His concept of oncogene addiction states that during the multistage carcinogenesis process, cancer cells become highly dependent on specific oncogenes. Furthermore, he distinguished oncogene mutations that occur early in the multistage process of tumor development because of their potential critical role in determining subsequent aspects of the abnormal circuitry in the evolving cancer cells. He also suggested that such early important mutations may well occur in the stem cell population of tumors. This interesting concept is directly applicable to targeted cancer therapy based on vital oncogenes [152,153,154].

The first significant study indicating the potential benefit of targeted cancer drug therapy directed against a fused oncogene was published by Druker [155]. Imatinib has revolutionized drug therapy for chromic myeloid leukemia (CML) and provided the groundwork for the development of a myriad of second and third generation drugs for CML, melanoma, kidney, as well as lung cancers [156,157]. The tremendous amount of cancer biology research over recent years has resulted in magnificent progress in the understanding of the molecular biology of the cancer cell. This has led directly to the unprecedented progress in the development of molecularly-targeted cancer therapies. Over the past few years, there has been a complete conceptual revolution in anticancer drug development. Unfortunately, this pantheon of targeted drugs has not been the panacea for the eradication of this disease. There remains a high failure rate, and few patients have a long-term survival benefit. Drug resistance is an increasingly common theme in targeted cancer treatment. Although Imatinib has transformed the approach to cancer therapy, even its long-term efficacy has been hindered by the development of drug resistance. This was a prelude to similar scenarios for virtually all of the subsequent driver oncogene-targeted drugs that have followed. It was first demonstrated in the case of Imatinib that the subsequent development of additional mutations in the region of the targeted drug binding site completely subverts the efficacy of the targeted drug [158,159]. This recurrent event is reflective of the role of such driver oncogenes in the ongoing cancer process. It is also probably reflective of the mutator phenotype of the cancer stem cell that results from the activity of vital oncogenes. Within human cells, there are innumerable regulatory mechanisms in place to accommodate changes in cellular homeostasis. These include feedback loops and crosstalk between the major signaling pathways. These mechanisms are ideal for a cell’s adjustment to varying dynamic physiological circumstances. Yet, these same mechanisms can wreak havoc on the efficacy of anticancer therapy that targets just the driver oncogenes and their pathways, leaving untouched the more vital oncogenes involved in cancer initiation and progression. The very nature of a cell mutator phenotype inevitably provides the mechanism for ongoing continuous and spontaneous mutations that lead to drug resistance in targeted driver oncogene pathways [160,161]. We must identify better targets.

Darnell pointed out the fact that transcription factors are overactive in most human cancer cells. He proposed that they are the most direct and hopeful targets for treating cancer [162]. Transcription factors that become overactive in cancers mediate the disproportionate transcription of genes that are required for tumor growth, progression and metastasis. Gene regulation is at the pinnacle of the cellular processes that determine normal cell function. It is primarily gene activation that is responsible for the ultimate transfer of genetic information within the cell, including that which determines cellular differentiation and proliferation. It therefore is not surprising that there has been appropriate interest in investigating transcription inhibition as a therapeutic modality in the effort to develop more effective targeted cancer therapy [163,164,165,166,167,168].

MicroRNAs play an important role in normal gene regulation. These small non-coding RNAs, measuring approximately twenty nucleotides, are involved in sequence-specific post-transcriptive gene silencing. They achieve this by base-pairing with complementary sequences in the 3’ untranslated regions of their targets. They are important partners of transcription factors. As a result, it is easy to see how they could contribute to a variety of biological processes that are driven by transcription, including tissue differentiation and cell proliferation. Since they are required for the fine regulation of transcription, the implication of this is that any dysfunction of microRNAs could significantly contribute to the cancer process [169,170,171,172,173]. For example, there may be a disruption of the processes that silence the transcription of transposable elements. MicroRNAs also play important roles in the regulation of cancer stem cell properties, including: asymmetric cell division, tumorigenicity and drug resistance [171]. There is the logical possibility that mutations affecting microRNA function are also a probable component of the progressive mutational events that result from the cell mutator phenotypic transformation orchestrated by the activity of vital oncogenes (Figure 1) [174,175,176]. MicroRNA mutations are associated with the worst outcome in some cancers [177]. The theoretical advantage bestowed upon vital oncogenes is a consequence of their novel and abnormal molecular structure as a result of their molecular pathological fused state. Unless evolutionary constraints have already established the presence of anticipatory complementary microRNA sequences to inhibit the abnormal chimeric messenger RNA transcript products of these super-oncogenes, potential inhibitory microRNA gene-regulatory processes would be completely undermined. The gene-regulatory inhibitor capacity of microRNAs that may hold for common-place driver oncogenes, such as *RAS*, becomes irrelevant. The discovery of RNA interference by Fire provided a potentially new method for interrupting gene function [178]. This method has been utilized in an attempt to target leukemic-specific fusion proteins [179]. The most challenging problem for the therapeutic application of siRNAs is the efficient delivery of siRNAs into leukemic tissues and specifically leukemic stem cells.

Because transcription activators usually do not have distinct areas of structural conformations, it becomes difficult to target specific binding sites on the protein’s surface. These are intrinsically disordered proteins that engage in many different protein-protein interactions during the formation of transcriptional complexes. However, this molecular biological fact allows the consideration of disrupting protein-protein interactions as a method of the targeted inhibition of vital oncogenes [180,181]. Erkizan utilized surface plasmon resonance screening to identify a lead compound that could block the binding of the transcription activator vital oncogene *EWS-FLI1* to its functional partner RNA helicase A. This resulted in the induction of apoptosis in Ewing’s sarcoma cells and reduced the growth of Ewing’s sarcoma orthotopic xenografts. Those results provided proof of principle that inhibiting the interaction of mutant cancer-specific transcription activators with the normal cellular binding partners required for their oncogenic activity provides a novel strategy for the development of unique effective tumor-specific anticancer drugs [182]. Grohar utilized high-throughput screening to identify his lead compound with activity against this same vital oncogene. That lead compound inhibited the expression of *EWS-FLI1* downstream targets at the mRNA and protein levels and decreased the growth of Ewing’s sarcoma cells in vitro. It also suppressed the growth of two different Ewing’s sarcoma xenograft tumors and prolonged the survival of Ewing’s sarcoma xenograft-bearing mice by causing a decrease in mean tumor volume [183]. In summary, these data indicate the realistic possibility of inhibiting the oncogenic consequences of the fused protein products of vital oncogene transcription activators.

### 8.2. Vital Oncogenes and Cancer Molecular Diagnostics

The initiation and progression of cancer is the direct result of genomic alterations. The revolution in genomic analysis has allowed the emerging concept of the individualized treatment and diagnosis of cancer [184]. The complexity, as well as the importance of gene fusions in cancer has become increasingly evident over the years. As a result, great efforts have been made to devise suitable diagnostic approaches that could help identify this crucial event in the cancer process [185,186,187,188]. DNA sequencing serves as the foundation for the elucidation of the numerous varieties of mutational events detected in cancer. Paired-end analysis has particular value as a result of its increased ability to map to a unique region of the genome and the ability to discover both small- and large-scale structural variations in the cancer genome [189,190]. Paired-end RNA sequencing may also be of some value [191]. Most importantly, it is the arrival of next generation sequencing (NGC) that has provided the legitimacy, accuracy and overall value to DNA sequencing in cancer diagnosis [192].

Traditionally, material for molecular diagnostic techniques has been obtained from tumor biopsies. It appears that circulating DNA is normally present in the blood and is seen at much higher levels in patients with cancer. The general belief is that these cancer-associated DNA fragments are the result of the apoptosis and necrosis of tumors. However, van der Vaart believed that DNA may be actively released by living cancer cells. He proposed that a disturbance of the equilibrium between the release of DNA by living cancer cells and the mechanisms involved in the clearing of this DNA may play the main role in the appearance of increased amounts of circulating DNA in the blood of cancer patients [193]. Regardless of the mechanisms involved, it has become increasingly clear that circulating cell-free nucleic acids can serve as important biomarkers in cancer patients [194,195]. The capacity to secrete cellular products is directly related to the phenomenon of cell-derived extracellular vesicles. Tatischeff has published an excellent review of the potential role of cell-derived extracellular vesicles in cancer [196]. Extracellular vesicles are more plentiful in cancers compared to their physiological counterparts. Moreover, these tumor-associated extracellular vesicles transport multiple functional molecular components, including DNA fragments [197]. Interestingly, mesenchymal stem cells are capable of producing cell-derived extracellular vesicles that promote angiogenesis [198]. Tumor-associated microvesicles have been found to contain not only amplified oncogene sequences, but transposons, as well [199]. Lee showed that rat epithelial cell transformation by the human H-ras oncogene leads to an increase in the production of small exosomal-like extracellular vesicles by the viable cancer cells [200]. These extracellular vesicles contained double-stranded full-length H-ras. Since detection of blood-borne genetic biomarkers in the cancer patient is a challenge because of the need for high sensitivity against the background of normal cellular DNA circulating blood, perhaps microvesicles released by tumor cells into the circulation will allow a greater accessibility to the genetic events that propagate the cancer. Is it possible that components of vital oncogenes are present, as well?

The detection of mutations in cell-free DNA from patients with cancer has been well established [201]. Most importantly, serial next generation sequencing of circulating cell-free DNA can be utilized for evaluating tumor response to molecular targeted drug therapy [202]. Perhaps a modification of these revolutionary techniques will provide the ultimate method for diagnosing and treating all cancers, since they all may well be a result of the presence of vital oncogenes [203].

## 9. Conclusions

Decades of accumulated cancer research data can now serve as the foundation for the construction of a logical molecular genetic model for cancer initiation and progression. This results in an entirely new paradigm for the molecular diagnosis of cancer and the application of targeted cancer therapy. It is assumed that certain mutations in cancer cells are vital to its initiation and early progression. Vital oncogenes are the fusion products of transcription activators that result in the production of oncogenic chimeric proteins with novel and wanton gene activating abilities. This results in the genomic dysregulation that transforms the affected normal tissue stem or progenitor cell in such a manner that its genome develops a mutator phenotype. The evolutionary heterogeneity of cancer is a mere by-product of the new mutational state of the cancer cell initiated by vital oncogenes. The paradigm presented here will hopefully allow us to focus more confidently on the identity of the genetic aberrations in cancer that are more relevant to the development of the molecular targeted individualized cancer therapies that could be curative.
